# Goal-Striving Stress and Bodily Pain among Working-Age Black and White Americans

**DOI:** 10.1007/s40615-025-02596-2

**Published:** 2025-08-07

**Authors:** Reed T. DeAngelis, Alexis C. Dennis, Sherrill L. Sellers, Harold W. Neighbors

**Affiliations:** 1 Institute for Social Research and Survey Research Center, University of Michigan, Ann Arbor, MI USA; 2 Department of Sociology and Centre On Population Dynamics, McGill University, Montreal, QC Canada; 3 Department of Family Science and Social Work, Miami University, Oxford, OH USA; 4 Department of Health Behavior and Health Equity, School of Public Health, University of Michigan, Ann Arbor, MI USA

**Keywords:** Black-White disparities, Goal-striving stress, Pain, Socioeconomic status, Subjective status, Stress process

## Abstract

This study tests novel research questions regarding Black-White disparities in bodily pain and goal-striving stress (GSS). A largely overlooked chronic stressor, GSS measures the psychosocial strains of blocked opportunity and dashed aspirations. We address our research questions among a socioeconomically diverse sample of Black and White working-age adults from Nashville, Tennessee (2011–2014; n = 1,252). We find that White adults with low socioeconomic status (SES) report the highest levels of GSS and pain, relative to their Black and high-SES White peers. Moreover, GSS predicts higher pain levels *only* among low-SES White adults. These patterns hold regardless of other sociodemographic characteristics, occupational hazards, chronic illnesses, stressful life events, and experiences with discrimination. Our findings have critical implications for researchers, clinicians, and policymakers concerned with rising rates of pain and opioid abuse among midlife adults in the United States. We discuss these implications and outline several avenues for future research.

**Supplementary Information:**

The online version contains supplementary material available at https://doi.org/10.1007/s40615-025-02596-2.

## Introduction

Black-White disparities in stress and health are widely documented. Compared to their White peers, Black Americans typically report higher levels of various chronic, acute, and anticipatory stressors [1–4]. Black Americans are also more likely to die prematurely from chronic illnesses like heart disease, diabetes, cancer, stroke, and kidney disease [5]. Relative to their Black peers, however, White Americans often exhibit higher rates of suicide and mental disorder [6], and tend to report worse mental health whenever they face similar psychosocial stressors [3, 7, 8].

Patterns like these have led some scholars to hypothesize that Black-White health disparities stem from group differences in stress-coping strategies [3, 8–10]. On the one hand, centuries of racial oppression have conditioned many Black Americans to remain hypervigilant to racism-related threats in their social environments [11]. While sometimes conducive to psychosocial resilience, high-effort coping with racism-related stressors can exhaust physiological systems over time, and eventually lead to poor physical health [12–14]. On the other hand, some scholars have argued that centuries of racial privilege have conditioned White Americans to overemphasize the role of individual agency and merit in achieving success [3, 9, 15]. Thus, when faced with inevitable setbacks, White Americans seem more inclined than Black Americans to internalize such experiences as personal failures, and suffer poor mental health as a consequence [3, 8, 9].

Recent studies have tested the stress-coping hypothesis with an underused scale of *goal-striving stress* (GSS) [3, 8, 16, 17]. Akin to other measures of subjective status [18], the GSS scale gauges the perceived gap between a person’s achievements and aspirations on a ten-rung ladder, and weights this gap by a stronger emotional attachment to aspirations and lower subjective likelihood of success [19, 20]. Someone with high GSS perceives a large achievement-aspiration gap and strongly desires to close this gap, but believes their chances for success are ultimately slim. Thus, the GSS scale is unique for emphasizing experiences of thwarted mobility due to social forces beyond one’s control [3]. Moreover, a growing body of research finds robust associations between GSS and a host of poor mental and physical health outcomes [3, 8, 17, 21–25].

One study found that GSS predicted worse mental health outcomes for White (vs. Black) adults [8]. Likewise, another study found that GSS predicted poor mental health only for White adults, but worse physiological health for Black adults [3]. A third study found that the links between GSS and poor physiological health were pronounced for Black adults who lived in predominantly White and affluent neighborhoods, where they experienced more frequent unfair treatment than Black peers who resided in predominantly Black and disadvantaged neighborhoods [16]. A fourth study of college-educated Black men found that those who attributed their GSS to systemic racism reported better mental health than their Black peers who did not make this attribution [17]. In short, the GSS literature supports the notion that Black-White health disparities could stem from differences in how both groups tend to anticipate and adapt to socioeconomic adversity.

One important question that remains unaddressed is whether GSS is implicated in Black-White disparities in *bodily pain*. This question is critical to answer for several reasons. First, bodily pain is a vital quality-of-life indicator that reflects a confluence of biopsychosocial processes [26]. Indeed, advances in the field of social neuroscience suggest that experiences associated with GSS, such as feelings of social failure or rejection, can generate chronic pain over time [27, 28]. To date, however, only one study has examined links between GSS and bodily pain [16]. But even this study did not consider Black-White disparities in GSS and its links to pain.

We also know very little about Black-White differences in the prevalence of bodily pain among the general population [29]. Most population-based studies focus on disparities in pain by socioeconomic status (SES), finding higher pain levels among lower-SES groups [30]. Relevant to GSS, some evidence also indicates higher levels of pain among groups who *perceive* themselves to be lower status, regardless of their objective SES [30, 31]. Other studies of clinical samples recruited from specialized pain management centers found that Black patients reported higher levels of clinical pain, lower levels of pain tolerance, and worse mental health than White patients [32, 33]. Some scholars have inferred from these findings that Black Americans are more vulnerable than White Americans to pain, due to the former group’s chronic exposures to racism [34]. However, national population-based studies have found either comparable levels of pain between Black and White adults [35], or higher levels for White adults, especially lower-SES Whites [36].

Given the current state of the literature, our study aims to address the following four open questions in this area of research: (1) Are there significant Black-White disparities in levels of GSS? (2) Are there significant Black-White disparities in levels of bodily pain? (3) Are Black and White Americans differentially vulnerable to the effects of GSS on pain? (4) Do any of these stress and pain processes further depend on individual SES? In the following sections, we report results from our analysis of a population-based sample of Black and White working-age adults from Nashville, Tennessee. We close by considering the research and policy implications of our findings.

## Methods

### Data

We addressed our research questions with survey data from the Nashville Stress and Health Study (NSAHS), a cross-sectional probability survey of US-born, non-Hispanic Black and White working-age adults who lived in Davidson County, Tennessee between 2011 and 2014 (N = 1,252). The NSAHS was collected in two stages. First, a simple random sample identified 199 block groups, from which 7,000 randomly selected addresses were sampled and screened. Of the households screened, 3,028 were deemed eligible. From these households, four stratified random samples were drawn of individuals aged 25 to 65, with equal representations of Black and White women and men. Fifty-nine percent of contacted persons ultimately agreed to participate in the study.

All interviews were computer-assisted and conducted in the respondent’s home or on the Vanderbilt campus. Interviewers were trained and matched to respondents based on race. Interviews lasted around three hours. Respondents received $50 for participating in the survey. To account for complex survey design, all analyses are weighted for the probability of selection during the household screening phase, and for nonresponse during the interviewing phase. Poststratification weights are also incorporated into the final design weight to permit generalizability to Davidson County’s population of Black and White working-age adults [37, 38]. The typical respondent is 44 years old, and reports 14 years of education and an annual household income of around $65,000. The sample also comprises slightly more women (n = 663) than men (n = 589), but almost equal numbers of Black (n = 627) and White (n = 625) respondents (see Table 1).

**Table 1:** Weighted Descriptive Statistics: NSAHS, 2011–2014

	White					Black							
	Low SES		High SES			Low SES			High SES			Total sample	
	(n = 196)		(n = 429)			(n = 430)			(n = 197)			(N = 1,252)	
Bodily Pain (range, 0–100)													
Past-month intensity	45.77	(31.80)	33.65	(28.88)	***	35.86	(36.02)	**	28.58	(30.10)	***	36.31	(31.58)
Past-month frequency	51.80	(38.98)	34.14	(32.78)	***	34.56	(38.56)	***	29.02	(33.41)	***	37.69	(36.27)
Past-month activity interference	35.45	(34.60)	21.35	(22.75)	***	24.38	(30.95)	***	16.51	(20.28)	***	24.63	(27.94)
Current intensity	16.55	(25.34)	9.17	(19.35)	***	14.13	(27.07)		5.76	(15.26)	***	11.44	(22.41)
Goal-Striving Stress (range, 0–100)													
Subjective distance from aspirations	31.78	(17.74)	18.71	(13.24)	***	30.18	(16.98)		23.79	(11.93)	***	24.26	(16.10)
Subjective low likelihood of attainment	47.29	(23.16)	35.59	(23.72)	***	39.65	(20.61)	***	35.20	(17.15)	***	38.94	(22.99)
Emotional attachment to aspirations	61.98	(28.51)	47.78	(30.25)	***	59.78	(32.39)		56.89	(27.62)		54.07	(30.73)
Socioeconomic Status (SES)													
Childhood financial status (range, 0–4)	2.28	(.92)	2.72	(.84)	***	2.17	(.94)		2.50	(.91)		2.50	(.92)
Education (years)	13.20	(2.38)	15.69	(2.94)	***	12.84	(2.68)		14.55	(2.54)	***	14.49	(3.02)
Personal income (1000 USD)	18.62	(14.11)	57.98	(35.96)	***	19.06	(12.68)		49.49	(24.60)	***	40.99	(33.61)
Household income (1000 USD)	34.36	(19.26)	91.89	(35.89)	***	26.28	(17.74)	**	76.29	(31.19)	***	65.21	(41.66)
Financial assets (1000 USD)	24.89	(77.85)	213.78	(289.22)	***	11.80	(16.24)		108.26	(180.30)	***	123.64	(233.37)
Value of home (1000 USD)	39.38	(69.25)	235.49	(191.68)	***	18.60	(45.28)	**	142.56	(168.24)	***	141.94	(178.36)
Covariates													
Female (vs. male)	.53	—	.49	—		.56	—		.52	—		.52	—
Age (in years)	42.77	(11.69)	45.44	(11.79)	*	43.47	(11.51)		43.79	(11.23)		44.32	(11.72)
Married (vs. single)	.44	—	.76	—	***	.29	—	**	.49	—		.57	—
Employment status													
Non-employed	.36	—	.15	—	***	.40	—		.07	—	***	.24	—
Non-hazardous job	.24	—	.58	—	***	.26	—		.54	—	***	.44	—
Hazardous job	.40	—	.27	—	**	.34	—		.39	—		.32	—
Diagnosed illnesses (count)	4.69	(3.47)	3.19	(2.67)	***	2.66	(2.43)	***	2.84	(2.62)	***	3.39	(2.92)
Major life events (count)	10.00	(5.54)	7.11	(4.59)	***	9.80	(5.10)		8.81	(5.34)		8.41	(5.15)
Lifetime discrimination (count)	1.05	(1.16)	.62	(1.00)	***	1.26	(1.53)		1.04	(1.26)		.88	(1.20)
Daily discrimination (scale)	2.14	(.58)	1.83	(.46)	***	2.19	(.67)		2.10	(.54)		1.99	(.56)

NSAHS = Nashville Stress and Health Study. Means/proportions are reported with standard deviations in parentheses. Estimates account for poststratification weighting and clustering at the block group level

^*^ p <.05, ** p <.01, *** p <.001 difference between low-SES Whites (two-tailed)

There are several reasons why the NSAHS sample is desirable for addressing our research questions. First, the NSAHS sampling strategy resulted in an equal representation of Black and White adults. Likewise, owing to the socioeconomic diversity of the Black population in Nashville, NSAHS researchers were able to oversample high-SES Black adults [37]. These features of the sample give us more leeway for stratifying our analyses by race and SES. Moreover, the NSAHS survey is one of few to include the GSS scale along with self-reports of bodily pain.

## Measures

### Bodily Pain

We measured bodily pain as a latent variable comprising four indicators. First, respondents were asked: “On average, how bad has your bodily pain been during the past four weeks?” Response options ranged from “no pain” (= 0) to “very severe” (= 5). Second, respondents were asked: “How often during the past four weeks have you had pain or discomfort?” Response options ranged from “no pain” (= 0) to “every day/almost every day” (= 5). Third, respondents were asked: “During the past four weeks, how many days did pain interfere with the things you usually do?” Response options ranged from 0 to 30 days. Fourth, respondents were asked: “How much bodily pain do you feel right now?” Response options ranged from “no pain” (= 0) to “very severe” (= 5). All items were rescaled to range from 0 to 100 with higher scores indicating more pain.

### Goal-Striving Stress

We measured GSS as a latent variable comprising three indicators [16]. Respondents were first shown an image of a ladder with rungs numbered from zero to nine. They were then told that “the steps on the ladder stand for the ten possible steps in your life. Level nine stands for the best possible way of life for you, and the first step stands for the worst possible way of life for you.” Respondents were then asked to report the rung they felt they currently occupied, the rung they aspired to reach in a few years, and their attitudes toward their aspired rung.

The first indicator is the respondent’s *subjective distance from aspirations*, calculated by subtracting the respondent’s achieved rung from their aspired rung (0 = no distance, 9 = maximum distance). Respondents who reported no achievement-aspiration gap were not asked any follow-up questions and were given a score of zero for all indicators (11% of the sample). The second indicator is the respondent’s *subjective likelihood of attainment* (1 = highly likely, 4 = highly unlikely). The third indicator is the respondent’s *emotional attachment to aspirations* (1 = unmet aspirations would not be disappointing at all, 4 = would be very disappointing). These indicators were also rescaled to range from 0 to 100. Higher scores capture respondents who feel further from their aspirations, more emotionally attached to their aspirations, but less optimistic about success.

### Race and Socioeconomic Status

We stratified our analytic sample into four groups according to respondents’ self-identified race and SES. The two race categories include US-born, non-Hispanic Black and White. SES is a composite measure of childhood financial status and adult education, personal income, household income, financial assets, and value of home. From these six items, we generated “high” and “low” SES groups based on the median of their first principal component score (eigenvalue = 3.02; shared variance = 0.50). We then combined respondent race and SES to create four groups of high- and low-SES Black and White adults.

### Childhood financial status

Was measured by asking respondents, “Which of these statements best describes your family’s financial situation most of the time while you were growing up?” Response options were ordinal and ranged from “could not afford to pay for food, clothing, and shelter” (= 1), to “could easily afford food, clothing, shelter, and lots of extras” (= 5). *Adult education* was measured in number of years. *Personal income* and *household income* were recorded on ordinal scales ranging from “none” (= 0) to “$135 k or more” (= 15). *Financial assets* included checking/savings accounts, and stocks/bonds/real estate (aside from home). Response options were ordinal and ranged from “less than $10 k” (= 0) to “more than $1 million” (= 7). *Home values* were recorded on an ordinal scale that ranged from “does not own a home” (= 0) to “more than $1 million” (= 8). For our descriptive analysis (Table 1), we recoded ordinal categories for income, assets, and home values to include the midpoint of each response in 1,000 USD (e.g., “$35 k-$45 k” = 40).

### Covariates

Our models included a set of covariates that could conceivably predict both GSS and bodily pain (i.e., confounders). We controlled for basic sociodemographic characteristics: *sex* (1 = female, 0 = male), *age* (in years), *marital status* (1 = married, 0 = single), and *employment status* (non-hazardous job, hazardous job, and non-employed). We coded respondents as having a *non-hazardous job* if they stated they were currently employed but they never were “in danger of injury or illness because of their job,” or never exposed to “a lot of noise or dirt on the job.” Respondents were categorized as having a *hazardous job* if they stated they were currently employed and exposed to the above hazards “once in a while,” “much of the time,” or “almost always.” These two groups were then compared to respondents who were currently *non-employed*.

We also controlled for additional stressors that might confound the association between GSS and pain. First, we included a 27-item checklist of *chronic illnesses* that lasted longer than a year and were diagnosed by a physician (e.g., heart disease, kidney disease, liver disease, ulcers, pancreatitis, migraines, etc.). Second, we included a 37-item checklist of *major life events* that could have occurred at any stage of the life course (e.g., abandoned by one or both parents, physically assaulted or mugged, had a spouse or partner die, etc.). Third, we controlled for a 7-item checklist of *lifetime discrimination* (e.g., unfairly fired or denied a promotion, unfairly treated by the police, landlord or realtor unfairly refused to sell or rent housing, etc.). Fourth and finally, our estimates accounted for a 9-item index of *daily discrimination* that averaged five-point Likert responses to items gauging daily and mundane episodes of unfair treatment (alpha = 0.85). Examples included being treated with less courtesy than others and being treated as unintelligent [39].

## Analytic Strategies

Our analyses proceeded in two steps. First, we examined basic descriptive statistics of all variables in our study, stratified across high- and low-SES Black and White groups (Table 1). The goal of this first phase of analysis was to determine whether there were any initial group disparities in levels of bodily pain and GSS. We used chi-square and t-tests to identify statistically significant differences in estimated means and proportions across groups. We chose low-SES White adults as the reference in our analyses because population studies have found high levels of pain among this group [36].

In the second phase, we tested a multigroup structural equation model (SEM) that estimated associations between latent variables of GSS and bodily pain, holding covariates constant. Our multigroup SEM estimated coefficients and standard errors separately for high- and low-SES Black and White groups. Clogg tests were then used to identify significant between-group differences in coefficients [40]. All models accounted for the complex design of the NSAHS by incorporating poststratification weights and clustering standard errors by block group [37].

The following variables had missing data: childhood financial status (n = 1), personal income (n = 17), household income (n = 31), financial assets (n = 17), value of home (n = 1), subjective distance from aspirations (n = 3), emotional attachment to aspirations (n = 1), major life events (n = 51), and daily discrimination (n = 8). To create our SES groups, we replaced missing observations for childhood financial status, personal/household income, assets, and value of home using 10 iterations of multiple imputation by chained equations [41]. Final SES scores include averages for each indicator across the ten imputed datasets. To estimate our multigroup SEM, we used full information maximum likelihood (FIML) methods to derive estimates and replace missing observations. For handling missing data in SEMs, simulation studies have found that FIML estimation is unbiased and more efficient than deletion and imputation methods [42].

Variable recoding and descriptive statistics were implemented in Stata 18. The multigroup SEM was tested in MPlus version 8. Our multigroup SEM exhibits acceptable fit to the data structure. The CFI and 1-RMSEA both score above the minimum accepted threshold of 0.90, at 0.92 and 0.94, respectively [43]. The BIC_k_ score is also − 1,303.11, indicating that our tested model provides a superior fit to the data than a fully saturated counterpart [44].

## Results

Weighted descriptive statistics of study variables are reported in Table 1. Separate columns are included for low- and high-SES Black and White adults. Reported p-values indicate statistically significant differences in means/proportions relative to low-SES Whites. Two key findings emerge from this table. First, low-SES Whites typically report the highest levels of pain symptoms. Second, with some exceptions, low-SES Whites tend to score higher than other groups on GSS indicators. For instance, while low-SES White and Black adults tend to report similar achievement-aspiration gaps and emotional attachments to their aspirations, low-SES Black adults typically report a higher subjective likelihood of success. Intriguingly, low-SES White adults also tend to report levels of discrimination and major life events that are comparable to Black respondents.

Table 2 reports the results from our multigroup SEM. Supplementary Table S1 reports results for our multigroup confirmatory factor analysis of latent variables. Table 2 reveals that GSS is positively associated with bodily pain only for low-SES Whites. Recall that pain and GSS are scaled from 0 to 100. Thus, a one-unit increase in GSS predicts a 0.82-unit increase in pain among low-SES Whites, holding covariates constant (b = 0.824; p < 0.01). Looking at standardized coefficients, the magnitude of this association is larger than any other predictor in the model (β = 0.319). According to a two-tailed Clogg test, the difference in coefficients for pain regressed on GSS is significantly larger than zero when comparing low-SES and high-SES Whites (z = 2.03). This same test between low-SES Whites and low-SES Blacks reaches one-tailed significance (z = 1.75), but is not statistically significant when comparing low-SES Whites with high-SES Blacks.

**Table 2:** Multigroup SEM of Recent Bodily Pain Symptoms: NSAHS, 2011–2014 (N = 1,252)

		Low-SES White			High-SES White					Low-SES Black					High-SES Black			
	**b**	**s.e**	**β**		**b**	**s.e**	**β**			**b**	**s.e**	**β**			**b**	**s.e**	**β**	
Goal-striving stress	.824	(.280)	.319	**	.192	(.135)	.091	†		.245	(.178)	.097			.326	(.214)	.112	
Age	.350	(.206)	.138		.127	(.133)	.058			-.060	(.328)	-.021			-.002	(.235)	-.001	
Female	5.973	(4.327)	.101		4.125	(2.579)	.081			9.674	(3.289)	.144	**		8.470	(3.609)	.157	*
Married	12.486	(4.003)	.209	**	4.789	(2.662)	.080			−1.121	(5.103)	-.015	†		5.548	(4.979)	.103	
Non-hazardous job ^a^	-.682	(6.787)	-.010		−6.092	(3.438)	-.118			−22.251	(4.476)	-.295	***	†	.534	(5.795)	.010	
Hazardous job ^a^	-.296	(6.099)	-.005		−1.655	(4.410)	-.029			−9.878	(6.703)	-.140			2.864	(3.616)	.052	
Major life events	.778	(.359)	.145	*	.523	(.327)	.094			.982	(.442)	.151	*		1.632	(.474)	.324	**
Diagnosed illnesses	2.146	(.617)	.252	**	2.979	(.555)	.311	***		3.414	(1.096)	.250	**		1.865	(.979)	.182	
Lifetime discrimination	1.400	(1.684)	.055		1.994	(1.171)	.078			1.332	(1.996)	.061			2.085	(2.013)	.097	
Daily discrimination	5.202	(3.501)	.101		.334	(2.949)	.006			4.790	(4.229)	.096			-.331	(3.521)	-.007	
Intercept	5.574	(11.768)	—		28.745	(5.080)	—	***		30.154	(6.975)	—	***		12.258	(6.612)	—	
R-squared	.420	(.063)	—	***	.206	(.046)	—	***	†	.271	(.041)	—	***	†	.305	(.077)	—	***
N	196				429					430					197			

Standardized (β) and unstandardized (b) weighted coefficients are reported with robust standard errors (s.e.) clustered by block groups in parentheses. SEM = structural equation model. Bodily pain and goal-striving stress are treated as latent variables. Covariates that are continuous or count variables are centered on their medians. Full information maximum likelihood techniques were used to derive estimates and impute missing values. The global fit of this model is as follows: Chi-square (df) = 485.618 (247). CFI =.92. 1-RMSEA =.94. BIC_k_ = −1,303.11

^a^ Reference = non-employed

^*^ p <.05, ** p <.01, *** p <.001 (two-tailed)

^†^ p <.05 difference between low-SES Whites (two-tailed)

## Discussion

Our study addressed four open questions regarding Black-White disparities in chronic stress and health. First, are there significant Black-White disparities in levels of goal-striving stress (GSS)? Second, are there significant Black-White disparities in levels of bodily pain? Third, are Black and White Americans differentially vulnerable to the effects of GSS on pain? Fourth, do any of these stress and pain processes further depend on individual SES? We examined these questions among a population sample of high- and low-SES Black and White adults from Nashville, Tennessee.

Our first key result is that low-SES White adults reported the highest levels of GSS, on average, followed closely by low-SES Black adults. Although past studies have investigated Black-White disparities in health outcomes linked to GSS, we still know surprisingly little about group differences in levels of GSS [3, 20, 45]. Recall that the GSS scale captures chronic anticipatory stress tied to fears of thwarted mobility strivings [3]. Our first finding suggests low-SES Whites felt especially discouraged about their current social status and prospects for upward mobility. Indeed, a key distinguishing feature between low-SES White and Black adults is that the latter group, despite perceiving themselves to be similarly low status, believed future success was more likely.

Our second key finding is that low-SES White adults also reported the highest levels of bodily pain. Moreover, GSS predicted increased pain *only* for low-SES Whites. These results have critical research and policy implications. First, they corroborate earlier population-based studies that found high rates of chronic pain among low-SES White adults [36]. Our findings also corroborate and extend previous research that found evidence to suggest GSS disproportionately affects the mental health of White (vs. Black) Americans [3, 8]. Our study ultimately advances the GSS literature by revealing further distinctions by SES and symptoms of bodily pain.

Why are low-SES White adults uniquely vulnerable to GSS and pain? An emerging research area on Whiteness and population health provides tentative answers [3, 9, 46, 47]. Accordingly, lower-status White Americans appear more inclined than other groups to unequivocally embrace ideologies of individualism and meritocracy, largely in efforts to distance themselves from the perceived inferiorities of minoritized racial-ethnic groups [46]. However, these same ideologies also appear to leave low-status Whites less prepared to cope with the harsh realities of socioeconomic adversity and blocked mobility [3]. Our study suggests that when faced with these realities, low-SES Whites interpret their lower status as indicative of personal failure and inferiority. Consistent with this claim, one aforementioned study found that White adults with higher GSS were more likely than their Black counterparts to report lower self-esteem [3]. Parallel insights from the field of social neuroscience indicate that chronic feelings of social rejection and inferiority can generate physiological pain symptoms in ways akin to physical stimuli like burns or cuts [27].

Another explanation focuses on the unique strengths and resiliencies of Black Americans [48]. For instance, Black adults typically report much higher levels of religious and spiritual involvement than their White peers [49]. Black Americans also tend to derive more psychosocial and health benefits from religious/spiritual involvement than White Americans [50, 51]. This appears to be especially true among low-SES Black adults [51]. Applied to our study, higher levels of religious and spiritual involvement could explain why low-SES Black adults tended to be more optimistic about success than their low-SES White counterparts, despite perceiving themselves to be equally low status. Future work should examine how religious/spiritual involvement interacts with perceptions of social status to affect the health of different racial-ethnic and SES groups.

Our findings may also have broader implications for parallel research on the so-called “Black-White mental health paradox.” This paradox refers to the fact that White Americans tend to exhibit worse mental health than Black Americans, as mentioned earlier, despite benefiting from centuries of racial privilege [52]. Previous studies have tested differential vulnerability to GSS as one potential explanation for the relatively poor mental health of White Americans [3, 8]. Our study advances this literature by showing how chronic pain linked to thwarted goal-strivings may contribute to poor mental health among White Americans, especially low-SES White adults.

The results of this study have important clinical implications. Clinical treatment lies at the end of a sequence of events under the control of the individual in pain. The perception of bodily pain is an important precursor for the decision to seek medical care, which then allows the clinical process to begin [53–55]. Our findings suggest that psychosocial stress associated with dashed aspirations can generate symptoms of bodily pain, especially for low-SES White adults. Clinicians should alert policy makers that subjective interpretations of broader social-structural conditions can underlie the pain that requires treatment [27, 28, 56].

Our study could also have policy implications for the alarmingly high and rising rates of morbidity and mortality observed among midlife adults in the United States (US) [57, 58]. When disaggregated by cause, fatal overdoses from synthetic opioids (e.g., fentanyl) appear to account for much of these trends [57]. Alongside rising rates of opioid abuse, reports of bodily pain have also steadily increased among the US working-age adult population in recent decades [59].

Researchers and policymakers typically rely on two explanations for these trends: supply-side and demand-side [57]. According to supply-side theories, deregulation of the pharmaceutical industry has made highly addictive and lethal prescription painkillers readily accessible to consumers. Thus, the recent rise in opioid-related deaths is attributed to the failure of government, public health, and medical institutions to protect consumers from corporate avarice [60]. According to demand-side explanations, macroeconomic shifts like post-industrialization and globalization have exacerbated socioeconomic inequality, generating increased chronic stress for lower-SES populations and, ultimately, greater demands for mind-altering substances [58].

Our study points to a possible demand-side explanation for rising rates of pain and opioid abuse among lower-status Whites: chronic psychosocial stress from thwarted aspirations, namely, GSS. For reasons we have largely speculated on, low-SES White adults seem uniquely vulnerable to the toxic effects of GSS on self-esteem, mental health, and bodily pain. There is a clear need for detailed inquiries into the underlying cultural worldviews and ideologies of White Americans, and how such belief systems interact with experiences and perceptions of socioeconomic adversity [9, 46]. Future research along these lines could reveal far-ranging insights into the experiences of low-SES White Americans, ranging from health and stress-coping to political behaviors [46, 61].

## Limitations and Future Directions

Our study has four key limitations that future studies should address. First, our findings apply only to the population of US-born, non-Hispanic Black and White working-age adults who lived in Davidson County, Tennessee between 2011 and 2014. Recent evidence suggests Black-White disparities in stress-coping and health vary across different regions of the United States [62]. Population health trends related to pain and opioid abuse, and related policies, have also changed over the last decade [57]. More research is needed to determine whether our findings generalize to other regions and time periods.

Second, the NSAHS data are cross-sectional and thereby preclude causal inference. Future work should replicate our findings with longitudinal data. One hypothesis worth investigating is whether GSS and bodily pain have reciprocal effects over the life course [63]. We could imagine, for example, that high levels of bodily pain may also cause someone to feel further removed from their ideal life and less optimistic about achieving future success. Still, researchers should ask whether and how different social groups are uniquely vulnerable to such reciprocal effects.

Third, the GSS scale is limited in certain respects. Future research may benefit from measuring mobility expectations across specific domains related to work, finances, etc. [64]. Likewise, measuring respondents’ reasons for why they may not reach their aspirations could be insightful. For instance, aforementioned work suggests that the mental health consequences of GSS might vary depending on whether individuals blame themselves or society for their struggles [17].

Fourth, our study did not examine gender variation. This is an important area for future research using larger population samples. Indeed, some evidence suggests that the multiplicative health effects of race, SES, and GSS may look different for Black and White women and men, who are known to face unique stressors and employ distinct coping strategies [50, 65–68].

## Conclusion

This study advances our understanding of the stress processes underlying Black-White disparities in bodily pain. Among a population sample of high- and low-SES Black and White adults from Nashville, we found that low-SES Whites reported the highest levels of goal-striving stress and pain. Low-SES Whites were also the only group to report positive associations between goal-striving stress and pain. These findings have critical implications for researchers, policymakers, and clinicians concerned with rising rates of pain and opioid abuse in the US. More research is needed to determine why low-status Whites appear uniquely vulnerable to goal-striving stress and pain.

## Supplementary Information

Below is the link to the electronic supplementary material.

Supplementary file1 (DOCX 15 KB)
