# Supplementary material for: Goal-Striving Stress and Bodily Pain among Working-Age Black and White Americans
Source: J Racial Ethn Health Disparities. 2025 Aug 7;13(6):4496–505. doi: 10.1007/s40615-025-02596-2 (PMC13367427; doi:10.1007/s40615-025-02596-2)
Supplement: Supplementary file 1 [file 40615_2025_2596_MOESM1_ESM.docx]

| **Table S1**. Multigroup Confirmatory Factor Analysis of Latent Variables: NSAHS, 2011-2014 (N = 1,252). | | | | | | | | | | | | | |
| --- | --- | --- | --- | --- | --- | --- | --- | --- | --- | --- | --- | --- | --- |
|  | White | | | | | |  | Black | | | | | |
|  | Low SES | | | High SES | | |  | Low SES | | | High SES | | |
|  | (n = 196) | | | (n = 429) | | |  | (n = 430) | | | (n = 197) | | |
|  | **b** | **s.e.** | **β** | **b** | **s.e.** | **β** |  | **b** | **s.e.** | **β** | **b** | **s.e.** | **β** |
| Goal-Striving Stress |  |  |  |  |  |  |  |  |  |  |  |  |  |
| Distance from aspirations | 1.000 | — | .675 | 1.000 | — | .915 |  | 1.000 | — | .787 | 1.000 | — | .743 |
| Emotional attachment to aspirations | 1.320 | (.160) | .510 | 1.580 | (.217) | .644 |  | 1.327 | (.181) | .521 | 1.656 | (.243) | .576 |
| Low likelihood of attainment | .944 | (.090) | .454 | 1.039 | (.120) | .537 |  | .761 | (.081) | .481 | .809 | (.128) | .442 |
| Rho reliability | .526 | — | — | .673 | — | — |  | .568 | — | — | .571 | — | — |
| Bodily Pain |  |  |  |  |  |  |  |  |  |  |  |  |  |
| Past-month intensity | 1.000 | — | .911 | 1.000 | — | .872 |  | 1.000 | — | .915 | 1.000 | — | .921 |
| Past-month frequency | 1.237 | (.036) | .939 | 1.164 | (.041) | .920 |  | 1.089 | (.034) | .936 | 1.086 | (.081) | .845 |
| Past-month activity interference | .868 | (.048) | .764 | .727 | (.036) | .811 |  | .775 | (.058) | .836 | .657 | (.045) | .867 |
| Current intensity | .501 | (.036) | .608 | .437 | (.042) | .583 |  | .562 | (.039) | .703 | .306 | (.049) | .500 |
| Rho reliability | .846 | — | — | .879 | — | — |  | .901 | — | — | .858 | — | — |
| Note: Unstandardized coefficients (b) are reported along with robust standard errors (s.e.) and standardized coefficients (β). Estimates account for poststratification weighting and clustering at the block group level. NSAHS = Nashville Stress and Health Study. | | | | | | | | | | | | | |
